# *MMRN1* as a Potential Oncogene in Gastric Cancer: Functional Evidence from In Vitro Studies and Computational Prediction of *NEDD4L*-Mediated Ubiquitination

**DOI:** 10.3390/cimb47110925

**Published:** 2025-11-06

**Authors:** Zhenghao Cai, Mengge Zhang, Qianru Zeng, Yihui Deng, Dingxiang Li

**Affiliations:** College of Traditional Chinese Medicine, Hunan University of Chinese Medicine, Changsha 410208, China

**Keywords:** gastric cancer, E3 ubiquitin ligase, prognostic signature, *MMRN1*, tumor microenvironment

## Abstract

Background: Gastric cancer (GC) remains a leading cause of cancer mortality. E3 ubiquitin ligases, as central regulators of protein stability and signaling within the ubiquitin–proteasome system, have been implicated in tumor progression, but their functional roles in GC are not well established. Methods: We integrated bioinformatics analysis of TCGA and GEO datasets, in vitro experiments (including cell proliferation, migration, and apoptosis assays), and computational modeling to identify key prognostic factors in GC. Results: We established two molecular subtypes (E3GC1/E3GC2) with distinct clinical outcomes and developed a 10-gene prognostic signature. The model showed moderate predictive accuracy (AUC: 0.61–0.71) and was validated externally. *MMRN1* was upregulated in GC cells and its knockdown significantly inhibited malignant phenotypes. Critically, drug sensitivity analysis revealed high-risk patients were more sensitive to proteasome inhibitors (bortezomib), while low-risk patients responded better to taxane-based chemotherapy (docetaxel). Molecular docking predicted a high-confidence interaction between *MMRN1* and *NEDD4L*, suggesting potential ubiquitination regulation. Conclusions: *MMRN1* drives GC cell proliferation and migration in vitro and may be regulated by *NEDD4L*-mediated ubiquitination. Our study provides a foundation for E3 ligase-based patient stratification and personalized therapy selection in GC. While this study provides comprehensive multi-omics evidence supporting the role of *MMRN1* in GC progression, its clinical translation is limited by the lack of in vivo validation and direct experimental evidence of *NEDD4L-MMRN1* physical interaction. Further studies using animal models and clinical specimens are warranted to confirm these findings.

## 1. Introduction

Gastric cancer (GC) stands as a malignant tumor with high prevalence, high invasiveness and substantial mortality [1]. Though early detection and targeted interventions help reduce GC-related deaths, the overall survival (OS) of individuals with GC is not favorable. Particularly, the 5-year survival rate of individuals with advanced GC is around 10%. The prognosis of individuals with distant metastases is even poorer, with a median OS of less than 10 months. Characterized by profound molecular heterogeneity and poor outcomes, GC persists as a main contributor to cancer-related fatality in the world despite multimodal therapies. Traditional biomarkers like TNM staging inadequately capture disease diversity [2]. The analysis by O Serra et al. and the 2025 NCCN Oncology Clinical Practice Guidelines emphasize that molecular typing and staging of gastric cancer can better reflect the diversity of the disease [3,4]. Hence, innovative models integrating post-translational modifications (e.g., ubiquitination) are required to optimize prognostication and guide precision interventions.

Ubiquitination, as a critical post-translational modification orchestrated by the ubiquitin–proteasome system (UPS), confers substrate specificity through E3 ubiquitin ligases, influencing cell cycle progression, metastasis, metabolism, and immune responses [5]. During ubiquitination, ubiquitin-activating (E1) and ubiquitin-conjugating (E2) enzymes prepare for the binding of ubiquitin, while ubiquitin ligases (E3) transfer ubiquitin to the lysine residues of target proteins [6]. Because E3 ubiquitin ligases confer substrate specificity for the binding of ubiquitin to target proteins, proteolysis-targeting chimera (PROTAC) drugs offer new treatment options [7].

Mounting evidence supports that prognostic signatures constructed based on E3 ligases are marked in GC. Identifying pivotal genes may reveal new molecular targets. Studies have reported that several E3 ubiquitin ligases, encompassing HECW1, FBXW2, and TRIM25, are linked to the invasion and metastasis, drug resistance, and formation of the immune microenvironment in GC [8,9,10]. Nevertheless, over 800 proteins with E3 enzyme activity have been established, and the role of distinct E3 enzymes in GC remains elusive [11]. Most previous studies focused on a single E3 ligase and lacked a systemic network perspective. Therefore, more systematic investigations are imperative to ascertain the role of E3 enzymes in GC.

In this study, E3-related genes were systematically identified from public databases by bioinformatics methods, and a novel gastric cancer risk prediction model was constructed based on these E3 ubiquitin ligases. The model was externally validated in an independent dataset from the GEO database. Further analysis showed that the model might serve as an independent prognostic factor, which was significantly correlated with a variety of tumor-related signaling pathways and involved in the regulation of tumor immune microenvironment (TIME). It is worth noting that Multimerin 1 (*MMRN1*), an E3-related gene closely related to poor prognosis, has not been fully studied in gastric cancer and has important research potential. Therefore, *MMRN1* was selected for in-depth analysis in this study to fill this knowledge gap and to evaluate its potential as a prognostic biomarker and therapeutic target.

## 2. Materials and Methods

### 2.1. The Source and Processing of Data

Transcriptomic data and clinical information on the TCGA-STAD cohort (412 tumors, 36 adjacent normals) were retrieved from UCSC Xena as training and internal validation datasets [12,13,14]. The expression data of the gene and clinical information from the GSE84433 dataset were retrieved from the GEO database for external validation [15]. We identified 898 E3 ubiquitin ligases from iUUCD 2.0 [16].

### 2.2. Differential Analysis and Consensus Clustering

Utilizing the limma package in R (version 3.62.2), DEGs between tumor and normal tissues in the TCGA-STAD cohort were confirmed in accordance with adjusted *p* < 0.05 and |logFC| > 2. Unsupervised consensus clustering analysis on E3 ubiquitin ligases was then implemented via the ConsensusClusterPlus package (version 1.17.0). Differential expression analysis was executed, leveraging the limma package for clustered molecular subtypes. With adjusted *p*-value < 0.05 and |logFC| > 1.5 as filtering criteria, E3-related DEGs (E3-DEGs) were confirmed across molecular subtypes.

### 2.3. Analysis of the Functional Enrichment

GO and KEGG functional enrichment analyses were implemented utilizing the clusterProfiler package in R (version 4.14.4). Prominent enrichment pathways were identified at *p* < 0.05. GSEA was conducted using the MSigDB Hallmark gene sets to analyze enrichment of E3-DEGs across gene sets.

### 2.4. Construction and Validation of a Prognostic Risk Model

Cox univariate regression was conducted to ascertain associations between E3-DEGs and OS of patients, identifying key prognosis-related E3DEGs (K-E3DEGs). TCGA-STAD data were split into training (70%) and test (30%) cohorts at random. A prognostic risk model was constructed leveraging K-E3DEGs through LASSO-Cox regression analysis, and the Cox proportional hazards model was fitted via the glmnet package (version 4.1.8) [17]. For LASSO-Cox regression, we applied 10-fold cross-validation with minimum criteria (lambda.min) to select the optimal penalty parameter. Risk scores were counted using the survminer (version 0.5.0) and survival (version 3.8.3) packages. As per the median risk score in the training cohort, participants were split into HR and LR cohorts. The distinctions in survival between the two cohorts were measured through Log-rank tests. External validation was performed using an independent GEO dataset. Kaplan–Meier curves were plotted, and *p*-values for survival distinctions between risk groups were calculated and displayed. To validate the proportional hazards assumption of the Cox regression model, we performed a Schoenfeld residuals test using the cox. zph function in the R survival package. A non-significant result (*p* > 0.05) for each variable and the global test indicated that the proportional hazards assumption was not violated.

### 2.5. Development and Verification of the Predictive Nomogram

A nomogram was developed leveraging the rms package (version 7.0.0) to estimate the probability of the disease and compute individual risk scores. Calibration curves (1/3/5-year) were leveraged to validate the consistency between predicted and actually observed survival rates. Time-dependent ROC curves were employed to appraise the predictive efficiency at different time points. Survival curves were leveraged to ascertain distinctions in survival between HR and LR individuals. The decision curve was leveraged to elucidate the clinical utility.

### 2.6. Immune Cell Infiltration Analysis

The abundances of 22 immune cell subtypes were quantified by applying the CIBERSORTx algorithm to deconvolute the normalized gene expression matrix. The CIBERSORT package (version 0.1.0) in R was run with default parameters to ascertain the proportions of immune cells, with cell types of zero abundance excluded. Stromal score, immune score, and estimate score were counted employing the Estimate algorithm. Distinctions in the proportions of immune cells between HR and LR cohorts were measured by the Kruskal–Wallis test, while group distinctions were visualized leveraging the ggplot2 package (version 3.5.1). ssGSEA analysis was implemented leveraging the GSVA package (version 2.0.5). The Kruskal–Wallis test was implemented to accommodate non-normally distributed data for assessing functional states of immune cells between HR and LR individuals.

### 2.7. Sensitivity Analysis of Drugs

The pRRophetic package (version 0.5.0) was leveraged to forecast IC50 values (half-inhibitory concentration) for 198 compounds in the tumor samples. Wilcoxon tests were utilized to identify drugs with significant distinctions in sensitivity analysis between risk groups (*p* < 0.001). Box plots were leveraged to present disparities in IC50 values between LR (blue) and HR (red) individuals, revealing the potential therapeutic effects of specific drugs for HR patients [18].

### 2.8. Cell Line Culture and Drug Treatment

GC cells of patients (AGS, MKN-45, MKN74, HGC-27) and normal gastric mucosal cells (GES-1) were obtained from the Cell Bank of the Chinese Academy of Sciences. Cells were rapidly resuscitated in a 37 °C water bath and subsequently cultured for expansion in Dulbecco’s Modified Eagle Medium supplemented with 10% fetal bovine serum. Culture was maintained at 37 °C in a humidified atmosphere containing 5% CO_2_ using an incubator. Subculturing was conducted using 0.25% trypsin-EDTA, with cells seeded at standardized densities. Transfection was performed using Lipofectamine 3000 (Thermo Fisher Scientific, Waltham, MA, USA) reagent to deliver either *MMRN1*-specific small interfering RNAs (si-*MMRN1*-1: 5′-CUGUAUCAAGUCUAUCAGAGG-3′; si-*MMRN1*-2: 5′-CCACUUUGACUGAUAUAGUGG-3′) or a negative control siRNA (siNC: 5′-ACGUGACACGUUCGGAGAA-3′). Cells were harvested at 48–72 h post-transfection for subsequent functional analyses. All experiments were performed in three independent replicates.

### 2.9. Quantitative Real-Time Reverse Transcription of PCR

Total RNA was extracted using TRIzol reagent. Cells cultured in 6-well plates were lysed with 1 mL TRIzol per well. After chloroform phase separation, the aqueous phase was collected, and RNA was precipitated with isopropanol, washed with 75% ethanol, and finally resuspended in nuclease-free water. RNA concentration and purity were determined using a microspectrophotometer. Reverse transcription was performed using a cDNA synthesis kit. A reaction mixture containing 1 μg RNA, 4 μL of 5× TRUE Reaction Mix, 1 μL of dsDNase, and 1 μL of Oligo(dT)_18_ primer was incubated at 37 °C for 60 min to synthesize cDNA.

Quantitative PCR (qPCR) was conducted using the SYBR Green method. The reaction system consisted of 10 μL of 2× SYBR Mix (High ROX), 0.4 μL of *MMRN1*-specific primers (forward: 5′-GGCATTGGGCTTAACAACAGT-3′, reverse: 5′-CGACATGACCCGAGTGGTT-3′), and 0.8 μL of cDNA template, with the final volume adjusted to 20 μL. Amplification was carried out on an ABI 7300 instrument under the following conditions: initial denaturation at 94 °C for 2 min; 40 cycles of 94 °C for 10 s and 60 °C for 30 s; followed by melt curve analysis (95 °C for 15 s, 60 °C for 1 min, and 95 °C for 15 s). GAPDH was used as the internal reference (forward: 5′-GAGTCAACGGATTTGGTCGT-3′; reverse: 5′-GACAAGCTTCCCGTTCTCAG-3′). Each sample was assayed in triplicate, and *MMRN1* expression was quantified using the 2^−ΔΔCt^ method. All experiments were independently repeated three times.

### 2.10. Western Blot Testing

Total cellular proteins were extracted using RIPA lysis buffer supplemented with 1% phenylmethylsulfonyl fluoride. The lysates were subjected to ultrasonication (60 Hz, 5 s per cycle, 5 cycles) and centrifuged at 12,000 rpm for 10 min at 4 °C. The supernatant was collected. Protein concentration was determined using a bicinchoninic acid (BCA) assay kit. Absorbance was measured at a wavelength of 562 nm using a microplate reader, and quantification was performed based on a standard curve. Equal amounts of protein (20 μg per lane) were denatured with 5× SDS loading buffer and separated by sodium dodecyl sulfate–polyacrylamide gel electrophoresis (SDS-PAGE) using a 10% stacking gel and a 15% separating gel. Electrophoresis was initiated at 80 V through the stacking gel and then continued at 100 V through the separating gel until the bromophenol blue tracking dye reached the bottom of the gel. Proteins were transferred to a polyvinylidene fluoride membrane (pre-activated with methanol for 20 s) using a wet transfer method at a constant current of 300 mA for a duration calculated as 1 min per kilodalton of target protein molecular weight. The membrane was then blocked with 5% skim milk powder in Tris-Buffered Saline with Tween-20 for 1 h at room temperature. Incubation with primary antibodies was performed overnight at 4 °C using an anti-*MMRN1* antibody diluted at 1:1000 in antibody dilution buffer. An anti-GAPDH antibody was used as an internal loading control. Following three washes with TBST, the membrane was incubated with a horseradish peroxidase (HRP)-conjugated secondary antibody diluted at 1:5000 for 1 h at room temperature. Protein bands were detected using an enhanced chemiluminescence substrate and visualized with a ChemiScope 6100 imaging system. Each experiment included three technical replicates and was independently repeated three times.

### 2.11. Cell Counting Kit-8

Cell proliferation was assessed using the Cell Counting Kit-8 (CCK-8) (Dojindo, Shanghai, China). The treated GC cells were seeded into a 96-well plate at a density of 3 × 10^3^ cells per well, with the peripheral wells filled with phosphate-buffered saline to minimize evaporation. Each experimental condition was performed in triplicate wells. After cell attachment, the culture medium in each well was replaced with fresh medium containing 10 μL of CCK-8 reagent. The plates were incubated at 37 °C in a 5% CO_2_ incubator for 2 to 4 h. The absorbance of each well was then measured at a wavelength of 450 nm using a microplate reader. Measurements were taken at 0, 24, 48, 72, and 96 h. Following each measurement, the medium containing CCK-8 reagent was aspirated and replaced with fresh complete medium. Cell viability was calculated according to the following formula: Cell viability (%) = [(OD_sample_ − OD_sterile_)/(OD_ontrol_ − OD_sterile_)] × 100%. A cell proliferation curve was generated by plotting time on the abscissa and the normalized absorbance values on the ordinate to evaluate the effect of *MMRN1* expression on cell proliferation. The entire experiment was independently repeated three times.

### 2.12. Colony Formation Assay

A colony formation assay was performed to evaluate the effect of *MMRN1* on the proliferative capacity of GC cells. HGC-27 cells in the logarithmic growth phase were harvested and dissociated with 0.25% trypsin. The cells were then seeded into 6-well culture plates at a density of 200 cells per well. Each well was filled with 2 mL of RPMI-1640 medium supplemented with 10% fetal bovine serum. The cells were cultured in a 37 °C, 5% CO_2_ incubator for 14 days, with the medium being replaced every 72 h. After the incubation period, the medium was aspirated. The cells were gently washed with PBS, fixed with 4% paraformaldehyde for 15 min at room temperature, and stained with 0.1% crystal violet for 15 min at room temperature. The plates were then rinsed gently under running tap water to remove excess stain. Colonies consisting of 50 or more cells were counted manually using an inverted microscope. Each experimental group was assayed in three independent replicates. The colony formation rate was calculated as (number of colonies/number of cells seeded) × 100%. Data are presented as the mean ± standard deviation. Statistical comparisons between groups were performed using an unpaired, two-tailed Student’s *t*-test, with a *p*-value of less than 0.05 considered statistically significant. To minimize evaporation-related edge effects, the peripheral wells of the culture plate were filled with sterile PBS.

### 2.13. Wound Healing Assay

A wound healing assay was performed to evaluate the effect of *MMRN1* on the migratory ability of GC cells. Forty-eight hours after transfection, HGC-27 cells were seeded into 6-well plates at a density of 5 × 10^5^ cells per well and cultured in a 37 °C, 5% CO_2_ incubator until approximately 70% confluence was reached. Three linear scratches per well were generated in the cell monolayer using a sterile 200 μL pipette tip. The detached cells and debris were removed by gently washing three times with PBS. The medium was then replaced with low-serum medium containing 1% FBS to minimize cell proliferation. Images of the wounds were acquired at 0 h using an inverted microscope at six predetermined fields per well. After 24 h of incubation, images were captured from the same fields. The migration distance was quantified by measuring the scratch width at six random points per scratch using the MRI Wound Healing Tool plugin in ImageJ software (version 1.54). The migration rate was calculated as follows: Migration rate (%) = [(Width at 0 h − Width at 24 h)/Width at 0 h] × 100. Each experiment was independently repeated three times. Data are presented as the mean ± standard deviation (SD). Statistical significance between groups was determined using an unpaired, two-tailed Student’s *t*-test, with a *p*-value of less than 0.05 considered statistically significant.

### 2.14. Transwell Migration Assay

The effect of *MMRN1* gene expression on the migratory capacity of GC cells was evaluated using a Transwell chamber assay. HGC-27 cells, harvested 48 h post-transfection, were detached by trypsinization, resuspended in serum-free RPMI-1640 medium, and adjusted to a density of 1 × 10^6^ cells/mL. A 100 μL aliquot of the cell suspension, containing 1 × 10^5^ cells, was seeded into the upper chamber. The lower chamber was filled with 700 μL of RPMI-1640 medium supplemented with 20% FBS to serve as a chemoattractant. The cells were subsequently incubated for 24 h at 37 °C in a humidified atmosphere containing 5% CO_2_. Following incubation, the Transwell chambers were rinsed gently with PBS. Cells that had migrated to the lower surface of the membrane were fixed with 4% paraformaldehyde for 15 min at room temperature and stained with 0.1% crystal violet for 15 min. Non-migratory cells remaining on the upper surface of the membrane were carefully removed using a cotton swab. For each membrane, five randomly selected non-overlapping fields were imaged with an inverted microscope under a 20× objective lens. The number of transmigrated cells (diameter >10 μm) was quantified using ImageJ software. Each experiment was performed in triplicate. The migration rate was calculated as follows: Migration rate (%) = (Mean number of migrated cells in the experimental group/Mean number of migrated cells in the control group) × 100. All data are presented as the mean ± standard deviation (SD) from three independent replicates. Statistical comparisons between groups were conducted using a two-tailed Student’s *t*-test, with a *p*-value < 0.05 considered statistically significant.

### 2.15. Flow Cytometric Analysis

The effect of *MMRN1* gene expression on apoptosis in HGC-27 cells was analyzed by flow cytometry using an Annexin V-fluorescein isothiocyanate (FITC)/propidium iodide (PI) apoptosis detection kit. Cells were harvested 48 h post-transfection. Subsequently, the cell monolayer was washed twice with PBS and detached by incubation with 0.25% trypsin at 37 °C until cell rounding was observed under an inverted microscope. Trypsinization was terminated by adding complete medium containing serum. The cell suspension was centrifuged at 300× *g* for 5 min to obtain a pellet. After centrifugation, approximately 1 × 10^5^ cells were resuspended in 195 μL of 1× binding buffer. Then, 5 μL of Annexin V-FITC and 10 μL of PI staining solution were added sequentially to the cell suspension. The mixture was vortexed gently and incubated for 15 min at room temperature in the dark. Stained samples were kept on ice and analyzed within 1 h using a flow cytometer. The instrument was configured with the following detection parameters: Annexin V-FITC was excited at 488 nm and emission was collected using a 525 nm bandpass filter; PI was excited at 488 nm and emission was collected using a 620 nm bandpass filter. Apoptotic cells were defined as follows: early apoptotic cells and late apoptotic cells. The total apoptosis rate was calculated as the sum of the early and late apoptosis rates. For each sample, a minimum of 10,000 cellular events were acquired. The experiment was independently repeated three times. Data are presented as the mean ± standard deviation (SD). The reagents used in this study are detailed in the Supplementary Table S1.

### 2.16. Molecular Docking

The E3 ubiquitin ligases targeting *MMRN1* with high confidence were screened from UbiBrowser database (v2.0). The target protein structure was predicted using AlphaFold2: *MMRN1* (UniProt ID: Q13201; AlphaFold model AF-Q13201-F1, 1228 amino acids) as the receptor (set to strand A), while the candidate E3 ligase *NEDD4L* was used as the ligand (set to strand B). The sequence number of the AlphaFold predicted structure of *NEDD4L* was AF-Q96PU5-F1, and the sequence number of UniProt was Q96PU5, with a total of 975 amino acids. Hybrid molecular docking (combining template-based modeling and free-docking algorithms) was performed on the HDOCK server for *MMRN1* and *NEDD4L*, resulting in a total of 1000 docking conformations. According to the docking score, the top models were selected for subsequent analysis. PyMOL (v2.5) was used to visualize hydrogen bonds and hydrophobic interactions at the protein–ligand interaction interface, and PDBePISA was used to quantify the interface interaction energy. To verify the specificity of the docking results, we set up negative control experiments in which *NEDD4L* was docked with an unrelated protein TRIM47 under the same parameters. In this control experiment, TRIM47 (human species, AlphaFold model AF-Q96LD4-F1, UniProt Q96LD4, 638 amino acids) was used as the receptor (chain A), while *NEDD4L* was used as the ligand (chain B). All protein structures were obtained directly from the AlphaFold database without preprocessing. Docking results were similarly visualized and analyzed for interaction using PyMOL and PDBePISA.

### 2.17. Statistical Analysis

Data analysis was conducted using R software (version 4.4.2) and GraphPad Prism software (version 9.0). Quantitative analysis of Western Blot bands and cell colony images was performed using ImageJ software. Statistical analysis strictly adhered to international standards: Student’s *t*-test or Mann–Whitney U test was used for comparison between two groups; one-way ANOVA or Kruskal–Wallis test was used for comparison among multiple groups; Kaplan–Meier method was used to draw survival curves for survival analysis, and the log-rank test with FDR correction was used to compare differences between groups, with multivariate analysis conducted using the Cox regression model; Pearson or Spearman methods were used for correlation analysis; Fisher’s exact test or χ^2^ test was used for categorical variables. The timeROC package (version 0.4.0) was used to calculate the area under the curve, and the consistency between predicted and observed values was evaluated through calibration curves. The clinical net benefit of the model was assessed using decision curve analysis. A *p*-value < 0.05 was considered statistically significant.

## 3. Results

### 3.1. Appraisal of E3 Ubiquitin Ligase-Based Molecular Subtypes of Gastric Cancer

**Figure 1:**
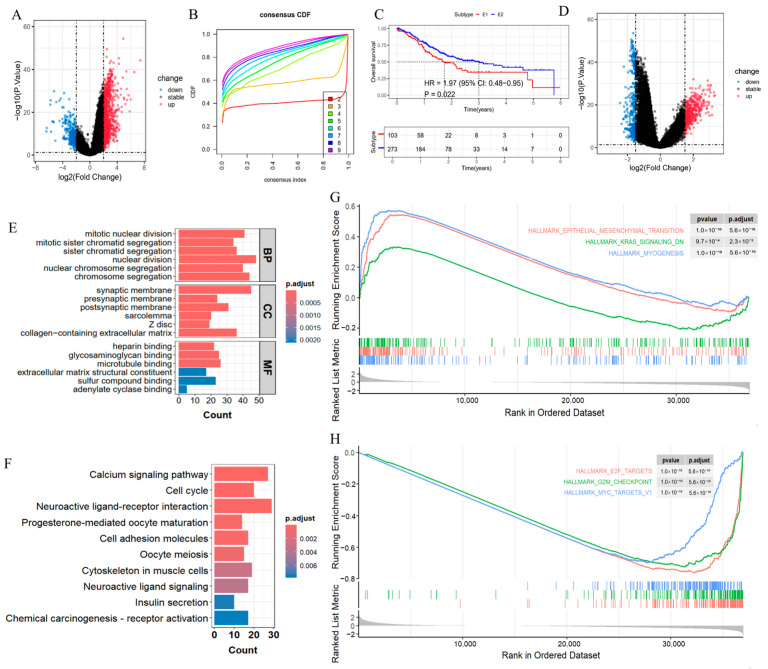
Consensus clustering based on E3 ligase in STAD. (**A**) Differential expression analysis between gastric cancer (GC) tissues and adjacent normal tissues using the limma package in R, with thresholds set at adjusted *p*-value < 0.05 and |logFC| > 2; (**B**) Unsupervised consensus clustering analysis performed using the ConsensusClusterPlus package to identify molecular subtypes based on E3 ubiquitin ligase expression profiles; (**C**) Kaplan–Meier survival curves generated to compare overall survival between subtypes, with statistical significance assessed using the log-rank test; (**D**) Differential expression analysis between E3GC1 and E3GC2 subtypes using the limma package, with thresholds set at adjusted *p*-value < 0.05 and |logFC| > 1.5; (**E**) Gene Ontology (GO) enrichment analysis conducted on differentially expressed genes between subtypes using the clusterProfiler package, with adjusted *p*-value < 0.05 as the significance threshold; (**F**) KEGG pathway enrichment analysis performed using the clusterProfiler package, with adjusted *p*-value < 0.05 as the significance threshold.; (**G**,**H**) Gene Set Enrichment Analysis (GSEA) of Hallmark gene sets (from MSigDB) to identify pathway activities between subtypes, using default parameters and adjusted *p*-value < 0.05 for significance.

E3 ubiquitin ligases are pivotal for normal cellular functions and hallmark pathways of cancer, highlighting their therapeutic potential in the treatment of cancers. We analyzed transcriptomic data and clinical information from patients with GC (STAD) in the TCGA cohort to delve into the heterogeneity in the expression of E3 ubiquitin ligases. Between TCGA gastric tumors and normal tissues, 1154 differentially expressed genes (DEGs) were determined (Figure 1A). Through unsupervised and consistent clustering analysis of a dynamic expression matrix of E3 ubiquitin ligases, we obtained two molecular subtypes associated with E3 ubiquitin ligase (E3GC1 and E3GC2) (Figure 1B). Survival analysis using TCGA-STAD data was implemented to elucidate the link of the E3GC1 subtype and E3GC2 subtype with OS. Patients with the E3GC1 subtype exhibited an inferior prognosis compared to those with the E3GC2 subtype (Figure 1C).

To delineate the biological disparities between the two clusters, differential analysis was first performed on E3 ubiquitin ligase-associated molecular subtypes (Figure 1D). Functional enrichment analysis revealed its potential functions. GO analysis demonstrated that the E3GC1 subtype exhibited heightened cell proliferation, aberrant activation of mitotic mechanisms, marked enrichment in collagen matrix pathways, and specific enrichment for cytoskeletal binding functions (Figure 1E). These findings indicated that compared to the E3GC2 subtype, E3GC1 displayed greater cell cycle dysregulation and marked fibrosis of the tumor microenvironment and was associated with enhanced cell motility. KEGG analysis (Figure 1F) revealed that compared to the E3GC2 subtype, E3GC1 showed marked enrichment in the neuroactive ligand–receptor interaction pathway, concomitant aberrant activation of both cell cycle and oocyte meiosis pathways, and the chemical carcinogenesis-receptor activation pathway. These findings indicated the presence of neural infiltration, disrupted cell division, and carcinogen receptor responses within the tumor microenvironment. GSEA revealed the activation of the epithelial–mesenchymal transition (EMT) pathway, sustained enhancement of myogenic differentiation programs, significant suppression of the KRAS signaling pathway, aberrant activation of E2F target genes, and activation of the G2/M checkpoint pathway. These alterations collectively drove cell cycle progression (Figure 1G), uncovering a cooperative EMT-cell cycle regulatory axis between molecular subtypes. EMT promoted tumor invasion and metastasis; E2F/G2M pathway activation accelerated cell proliferation, and KRAS suppression may contribute to treatment resistance.

### 3.2. Establishing an E3 Ubiquitin Ligase-Associated Prognostic Signature in GC

**Figure 2:**
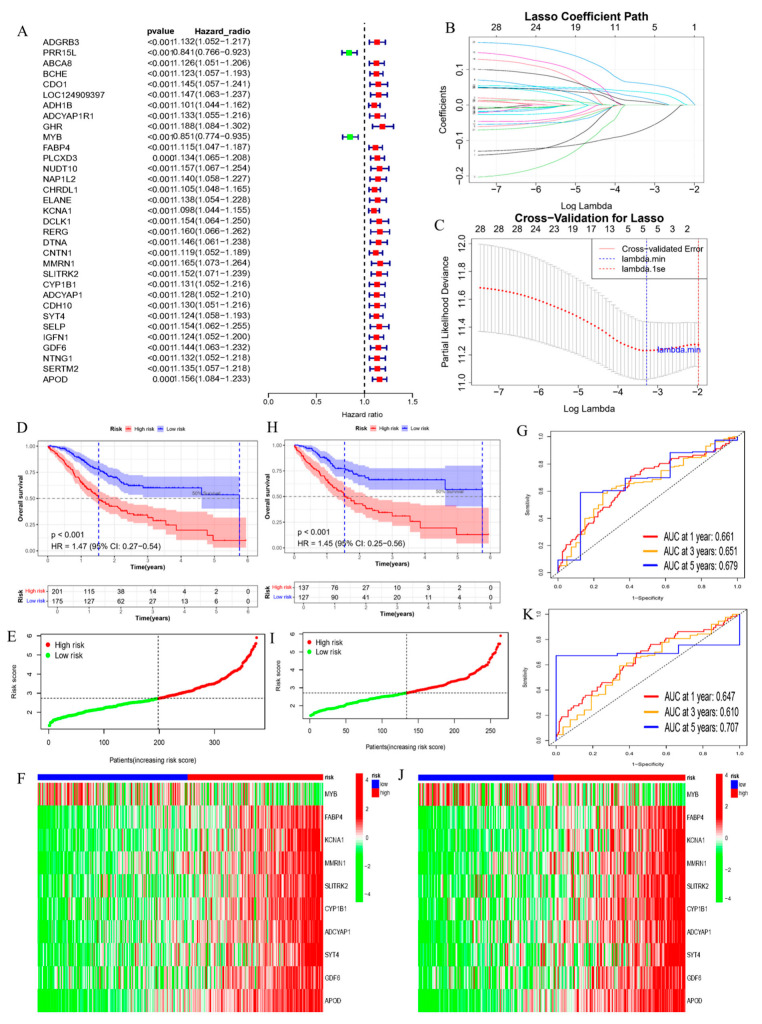
Establishment of an E3 ligase-related prognostic signature model in STAD. (**A**) Univariate Cox proportional hazards regression analysis performed on E3-related differentially expressed genes (E3DEGs) between molecular subtypes to screen for genes with prognostic significance (*p*-value < 0.001); (**B**) LASSO coefficient plot generated using the glmnet package in R, showing the trajectory of coefficients as a function of Log(λ). Gray vertical lines indicate the standard error of cross-validation error at each λ, with dashed lines corresponding to λ.min and λ.1se; (**C**) Parameter selection in the LASSO model, displaying the change in regression coefficients with Log(λ). Each curve represents a variable’s coefficient, with vertical lines marking λ.min and λ.1se positions; (**D**) Kaplan–Meier survival analysis conducted on the complete dataset using the survival package in R, comparing high-risk (HR) and low-risk (LR) groups; (**E**) Distribution of risk scores calculated based on the prognostic signature formula in the complete dataset; (**F**) Heatmap of gene expression profiles for the 10 prognostic genes, stratified by risk group (HR vs. LR), generated using the pheatmap package (version 1.0.12); (**G**) Time-dependent ROC curves plotted using the timeROC package to assess the predictive accuracy of the risk score for 1-, 3-, and 5-year overall survival in the complete TCGA-STAD dataset; (**H**) Kaplan–Meier survival analysis performed on the training set (70% of data) to compare HR and LR groups; (**I**) Distribution of risk scores in the training set; (**J**) Heatmap of gene expression in the training set, showing expression patterns of the 10 prognostic genes across risk groups; (**K**) Time-dependent ROC curves for the training set, evaluating the risk score’s performance in predicting 1-, 3-, and 5-year survival.

Univariate Cox analysis was implemented on DEGs between the two molecular subtypes (E3DEGs) to select genes with significant prognostic value. At a significance level of *p* < 0.001, 23 genes associated with poor prognosis (HR > 1.0) and 2 protective genes (HR < 1.0) were identified (Figure 2A).

The TCGA-STAD cohort was separated into training (70%) and testing (30%) sets at random. Within the training set, 25 survival-associated E3 ubiquitin ligase-related DEGs were put into the LASSO-Cox regression model, and the Cox model was fitted leveraging glmnet. Optimal lambda was selected via 10-fold cross-validation (Figure 2B,C), and 10 prognostic genes (*MMRN1*, *APOD*, *SLITRK2, ADCYAP1*, *KCNA1*, *GDF6*, *CYP1B1*, *FABP4*, *MYB*, and *SYT4*) were identified. Risk scores were calculated, and a threshold was determined (Figure 2E,I). The log-rank test was leveraged to ascertain survival distinctions between risk groups. The result unveiled that high-risk (HR) individuals had a worse clinical outcome than low-risk (LR) individuals (Figure 2D,H). The expression levels of 10 prognostic genes in the HR and LR cohorts are depicted in Figure 2F,J. The risk score formula constructed by these 10 genes was as follows: RiskScore = (0.0333 × *MMRN1*) + (0.0591 × *APOD*) + (0.0207 × *SLITRK2*) + (0.0144 × *ADCYAP1*) + (0.0124 × *KCNA1*) + (0.0013 × *GDF6*) + (0.0011 × *CYP1B1*) + (0.0199088139628989 × *SYT4*) + (0.0005 × *FABP4*) − (0.0157 × *MYB*).The AUC values for prognosticating 1-, 3-, and 5-year survival were 0.64, 0.61, and 0.71, respectively (Figure 2G,K). Furthermore, Schoenfeld residual tests were performed to evaluate the proportional hazards assumption of the Cox model. The results indicated that the proportional hazards assumption was not violated for any of the variables included in the final model, confirming the structural validity of the fitted model. The results are summarized in Supplementary Figure S1.

### 3.3. Validation of the E3 Ubiquitin Ligase-Associated Prognostic Signature for GC (STAD)

**Figure 3:**
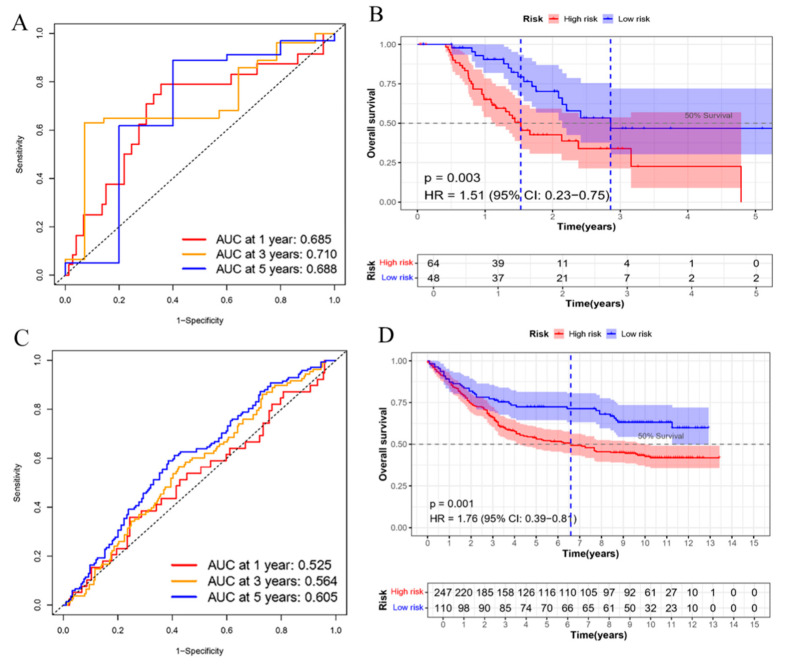
Validation of the E3 ubiquitin ligase-related prognostic signature model in STAD. (**A**) Time-dependent ROC curves generated for the TCGA-STAD test set using the timeROC package in R, to evaluate the predictive performance of the risk score signature for 1-, 3-, and 5-year overall survival; (**B**) Kaplan–Meier survival analysis performed on the TCGA-STAD test set using the survival package in R, comparing high-risk (HR) and low-risk (LR) groups based on the risk score stratification; (**C**) Time-dependent ROC curves plotted for the external validation cohort (GSE84433) using the timeROC package, to assess the prognostic accuracy of the risk score for 1-, 3-, and 5-year survival; (**D**) Kaplan–Meier survival analysis conducted on the GSE84433 cohort using the survival package, comparing HR and LR groups.

The robustness of the E3 ligase-based prognostic signature was verified in the TCGA-STAD testing set and an external cohort. In the TCGA-STAD testing set, time-dependent AUC values of the risk score for forecasting 1, 3-, and 5-year survival were: 0.69, 0.71, and 0.69, respectively (Figure 3A,B). Within the GSE84433 cohort, AUC values forecasting 1, 3-, and 5-year survival were 0.525, 0.564, and 0.605 (Figure 3C,D). The prognostic model had moderate predictive accuracy. Similarly, external validation in the GSE84433 cohort showed limited applicability and possible overfitting. Although this model provides a useful tool for risk stratification, its clinical application needs further refinement and validation in a larger cohort.

### 3.4. Development of a Prognostic Nomogram for GC Survival

**Figure 4:**
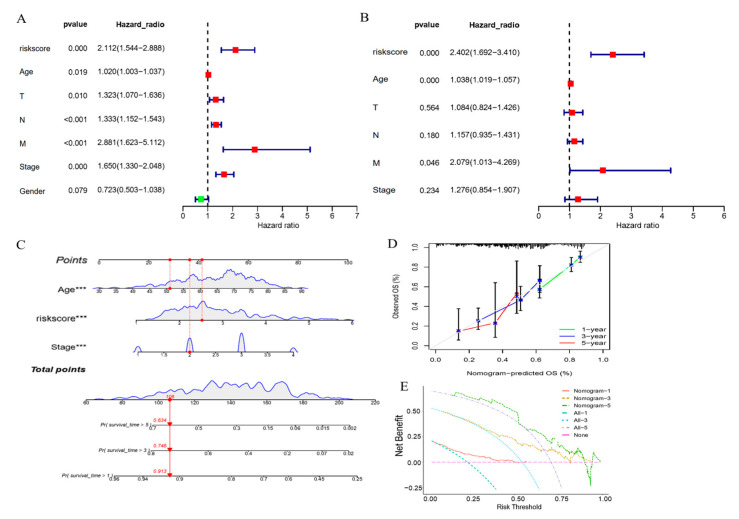
Nomogram for prognosticating the survival in STAD. (**A**) Univariate Cox regression analysis performed on clinical features (including age, gender, and TNM stage) and risk scores in the TCGA-STAD dataset using the survival package in R; (**B**) Multivariate Cox regression analysis conducted to assess the independent prognostic value of risk scores and clinical factors, using the Cox proportional hazards model implemented in the survival package; (**C**) Construction of a nomogram integrating age, risk score, and tumor stage to predict 1-, 3-, and 5-year survival probabilities. The nomogram was developed using the rms package in R, with variable axes scaled based on regression coefficients. The (***) after the variable name in the nomogram indicates that these variables are statistically significant (*p* < 0.001) in the multivariate Cox proportional hazards model; (**D**) Calibration curves generated to evaluate the agreement between predicted and observed survival probabilities at 1, 3, and 5 years, using the rms package with bootstrap resampling; (**E**) Decision curve analysis performed to assess the clinical utility of the nomogram by comparing net benefits across different risk thresholds (0–1.0), using the rmda package in R (version 1.6). Reference strategies included “All” (treat all patients) and “None” (treat no patients).

Univariate and multivariate Cox regression analyses disclosed that the prognostic score related to E3 ligase served as an independent prognostic predictor for individuals with GC, unaffected by age, pathological staging, or tumor staging (Figure 4A,B). The risk score retained high significance in the multivariate model (HR > 1, *p* < 0.001). Its effect size was enhanced in the optimized model, where T/N/M staging was excluded from the significant variables (*p* > 0.05), indicating that its prognostic information was efficiently integrated by the risk score. This model markedly streamlined clinical decision-making while improving prognostic predictive efficacy. Subsequently, a nomogram was constructed to augment the performance of the E3 ubiquitin ligase-based risk model, providing a quantitative visual tool for forecasting the 1-, 3-, and 5-year OS (Figure 4C). Furthermore, calibration curves unraveled that the predictive performance of the nomogram was satisfactory (Figure 4D). Decision curve analysis (DCA) also unveiled that the nomogram had a notable net benefit for the prediction of the mortality risk, outperforming conventional age and TNM staging systems (Figure 4E). These findings disclosed that the nomogram model had important clinical value in forecasting survival in individuals with GC.

### 3.5. E3 Ubiquitin Ligase-Based Risk Stratification Reveals the Heterogeneity of the Immune Microenvironment

**Figure 5:**
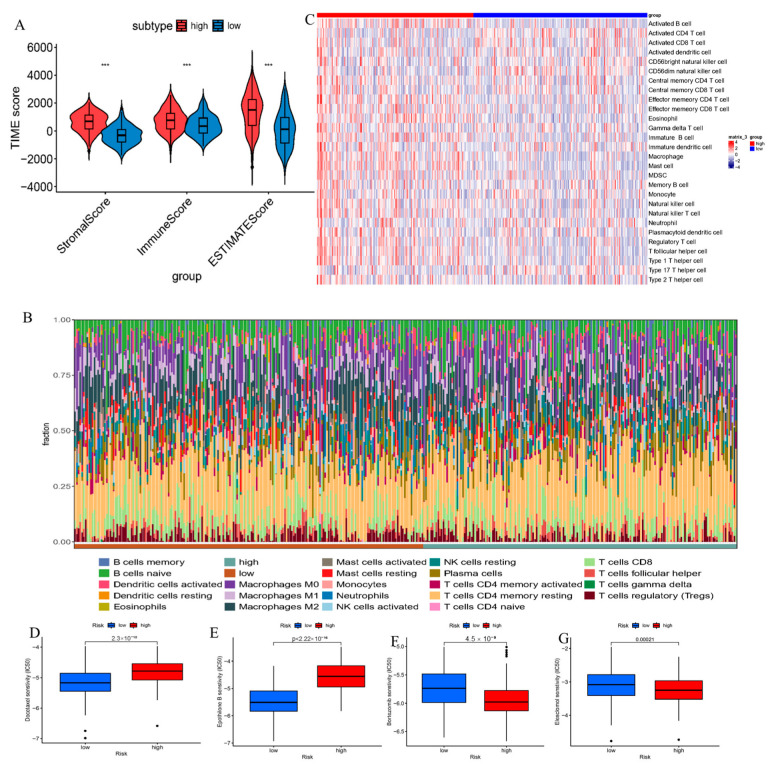
Distinctions in immune microenvironment diversity and anti-cancer drug sensitivity between HR and LR individuals as per E3 ubiquitin ligases. (**A**) Comparison of TIME scores (stromal, immune, and ESTIMATE scores) between HR and LR groups using the ESTIMATE algorithm implemented in R; (**B**) Immune cell composition analysis inferred via the CIBERSORTx algorithm, deconvoluting the normalized gene expression matrix to quantify the proportions of 22 immune cell subtypes. Significance annotations: *** (*p* < 0.001); (**C**) Functional activity scores of immune cells assessed using single-sample gene set enrichment analysis (ssGSEA) with the GSVA package in R (version 1.52.0), based on MSigDB Hallmark gene sets. (**D**–**G**) Drug sensitivity analysis illustrated via IC_50_ values for four compounds (docetaxel, eribulin, bortezomib, elesclomol), calculated using the pRRophetic package in R with default parameters.

The ESTIMATE algorithm unveiled that stromal, immune, and ESTIMATE scores in HR individuals were notably elevated in comparison to LR individuals (*p* < 0.001). This finding showed that the tumor microenvironment of HR individuals exhibited an elevated level of stromal and immune cell infiltration, suggesting an immune activation state (Figure 5A). Given the heterogeneity in TIME between the two cohorts, CIBERSORTx deconvolution was implemented to measure the proportion of 22 immune cell subsets in STAD. The result implied that in HR individuals, naïve B cells, M2 macrophages, resting mast cells, and monocytes (*p* < 0.05) were notably enriched. Conversely, LR individuals exhibited higher proportions of M0 macrophages, activated mast cells, neutrophils, resting NK cells, and resting CD4+ memory T cells (*p* < 0.05) (Figure 5B). HR individuals exhibited immunosuppression and an inactive immune state in their TIME, which may promote tumor immune escape. Nonetheless, LR individuals showed stronger immune activity and potential anti-tumor immunological memory.

The activity scores of immune cells in HR and LR individuals were compared via ssGSEA utilizing the GSVA package. Elevated activity was identified in HR individuals for 15 cell types, including activated B cells, central memory CD8 T cells, and effector memory CD4/CD8 T cells, in comparison to LR individuals. On the contrary, activated CD4 T cells, CD56dim NK cells, and Th17 cells demonstrated higher activity in LR individuals (Figure 5C). HR individuals demonstrated widespread immunosuppression and exhaustion, accompanied by abnormal activation of B cells and memory T cells, reflecting tumor-mediated immune dysregulation. LR individuals showed a more robust cytotoxic and pro-inflammatory immune response, potentially contributing to anti-tumor immunity.

### 3.6. Differences in the Sensitivity to Anticancer Drugs Between High-Risk and Low-Risk Cohorts Based on E3 Ubiquitin Ligase

**Figure 6:**
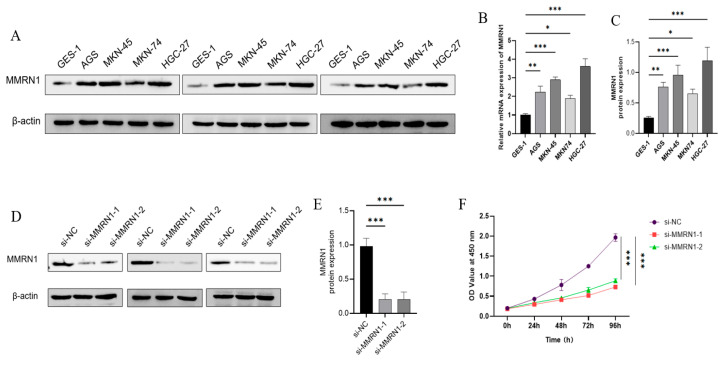
Oncogenic role of *MMRN1* through promoting proliferation in gastric cancer cell. (**A**) Western blot analysis of *MMRN1* protein expression in gastric cancer cell lines (MKN74, AGS, MKN-45, HGC-27) and normal gastric mucosal cells (GES-1), performed using standard protocols with GAPDH as loading control; (**B**) qRT-PCR analysis of *MMRN1* mRNA expression in gastric cancer cell lines and GES-1 cells, using GAPDH as internal reference and the 2^−ΔΔCt^ method for quantification. Significance annotations: * (*p* < 0.05), ** (*p* < 0.01), *** (*p* < 0.001); (**C**) Western blot validation of *MMRN1* protein expression in the same cell lines. Significance annotations: * (*p* < 0.05), ** (*p* < 0.01), *** (*p* < 0.001); (**D**,**E**) Assessment of *MMRN1* knockdown efficiency via Western blot after transfection of HGC-27 cells with two siRNAs (si-*MMRN1*-1 and si-*MMRN1*-2), using si-NC as negative control. Significance annotations: *** (*p* < 0.001); (**F**) CCK-8 cell proliferation assay conducted after transfection, measuring optical density at 450 nm at 0, 24, 48, 72, and 96 h to assess cell viability. Significance annotations: *** (*p* < 0.001).

For distinctions in the sensitivity to anticancer drugs among different risk groups, patients with cancer in different risk groups responded differently to several treatments, which may contribute to the failure of treatment and the recurrence of the tumor. We counted the values of the half-maximal inhibitory concentration (IC50) for each drug in the TCGA-STAD dataset samples. The sensitivity of the drug in patients with GC (STAD) was analyzed. The analysis unraveled that LR individuals demonstrated higher sensitivity to docetaxel and epirubicin (Figure 6D,E). Additionally, HR individuals demonstrated markedly higher sensitivity to bortezomib, ipilimumab in contrast to LR individuals (Figure 5F,G).

### 3.7. MMRN1 Knockdown Suppresses the Progression of GC

Among the candidate prognostic genes, the roles of APOD, SLITRK2, ADCYAP1, KCNA1, GDF6, CYP1B1, FABP4, MYB, and SYT4 in the progression of GC have been tested in prior studies [19,20,21,22,23,24,25,26,27].Therefore, we focused on the *MMRN1* gene, which was ranked high in the risk score of the GC prognostic model established in this study. Moreover, functional mechanistic studies on its role in GC are relatively scarce. To examine the effect of *MMRN1* in GC cells, we quantified its levels in GES-1, AGS, MKN-45, MKN74, and HGC-27 using RT-qPCR and Western blot. *MMRN1* was markedly upregulated across all four GC cells compared to normal cells, demonstrating progressively increasing expression levels (Figure 6A). Its expression level was the lowest in normal cells, markedly elevated in cancer cell line 1, further increased in MKN74 and MKN-45, and was the highest in HGC-27 (Figure 6B,C). This trend suggested that *MMRN1* may be closely tied to the development and progression of GC, likely driving tumorigenesis or sustaining malignant traits.

Subsequently, siRNA was leveraged to inhibit the expression profile of *MMRN1* in the HGC-27 cell line. RT-qPCR and Western blot were utilized to validate the testing results of *MMRN1* and the protein level of the housekeeping gene GAPDH. Results indicated that the band of *MMRN1* was enhanced in MKN74, AGS, MKN-45, and HGC-27 cell lines compared to normal controls, confirming upregulated protein expression. This protein expression trend aligned with qPCR results, demonstrating that transcriptional upregulation of *MMRN1* was consistently translated to elevated protein expression. Furthermore, both si-*MMRN1*-1 and si-*MMRN1*-2 effectively suppressed the expression of *MMRN1* in HGC-27 cells versus si-NC controls (Figure 6D,E).

CCK8 assays were implemented in the HGC-27 cell line with *MMRN1* knockdown. The results unraveled that the OD 450 nm absorbance values at 24, 48, 72, and 96 h were notably lower in the si-*MMRN1*-1 and si-*MMRN1*-2 groups compared to the si-nc control group, indicating that *MMRN1* silencing effectively inhibited cell activity. These findings implied that the expression of *MMRN1* was strongly linked to the proliferation of GC cells (Figure 6F).

**Figure 7:**
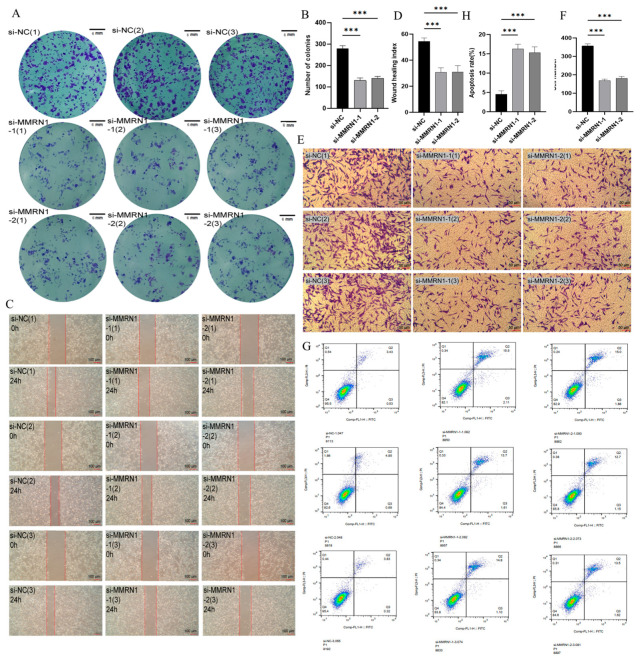
Knockdown of *MMRN1* suppresses the progression of GC cells. (**A**) Colony formation assay performed on HGC-27 cells after transfection with si-*MMRN1* (si-*MMRN1*-1 and si-*MMRN1*-2) or negative control siRNA (si-NC). Cells were stained with crystal violet after 14 days of culture; (**B**) Quantitative analysis of colony numbers from the colony formation assay. Significance annotations: *** (*p* < 0.001); (**C**) Wound healing assay conducted on HGC-27 cells after transfection. Scratch wounds were generated using a pipette tip and imaged at 0 and 24 h. Red dashed lines indicate initial wound boundaries; (**D**) Quantitative measurement of wound closure rate from the wound healing assay, calculated as the percentage of wound area reduction after 24 h using ImageJ software. Significance annotations: *** (*p* < 0.001); (**E**) Transwell invasion assay performed using Corning chambers with Matrigel coating. Cells were stained with crystal violet after 24 h of invasion; (**F**) Quantitative analysis of invaded cells from the Transwell assay. Significance annotations: *** (*p* < 0.001); (**G**) Flow cytometry analysis of apoptosis using Annexin V-FITC and PI staining after transfection. The Q2 quadrant represents early apoptotic cells; (**H**) Quantitative analysis of total apoptosis rate. Significance annotations: *** (*p* < 0.001).

After interfering with the expression of *MMRN1*, the proliferation capabilities of HGC-27 cells were substantially inhibited (Figure 7A,B). In addition, the Transwell experiment disclosed that abundant cells in the si-nc group migrated across the chamber membrane to the lower chamber, while the number of cells that crossed the membrane considerably declined in the si-*MMRN1*-1 and si-*MMRN1*-2 groups. This result unveiled that *MMRN1* may exert a pivotal role in boosting the migration of GC cells, and its knockdown can effectively block the migration of cells (Figure 7E,F). Wound healing assays further validated the impact of *MMRN1* on the migration capacity of GC cells. Identical scratch widths were generated across all groups at 0 h. After 24h, cells in the si-nc group displayed a robust migration ability with near-complete wound closure, while the si-*MMRN1*-1 and si-*MMRN1*-2 groups were only partially healed. This observation confirmed that *MMRN1* silencing markedly impaired the migration of GC cells (Figure 7C,D). Furthermore, flow cytometry analysis demonstrated that inhibiting *MMRN1* notably promoted apoptosis in GC cells (Figure 7G,H). These in vitro experimental findings suggest that inhibition of *MMRN1* may slow down the malignant phenotype of GC cells.

### 3.8. Molecular Docking Analysis of MMRN1 with Upstream Regulators

Molecular docking results indicated a potential ability to form a stable complex between *MMRN1* and E3 ligase *NEDD4L*. Among the 1000 docking conformations generated by the HDOCK server, the top 10 models with docking scores are shown in Table 1. Among them, model 1 showed the best docking score (−351.90) and the highest confidence (0.9827), indicating that this conformation was the most reliable at both the structure and energy levels, and was selected as the object of subsequent analysis.

In-depth analysis of this optimal model (Model 1) revealed (Table 2) that the complex of *MMRN1* and *NEDD4L* had a significantly negative binding free energy (ΔG = −10.4 kcal/mol), suggesting that the binding between the two is a spontaneous process. The complex has a large interface area and involves a total of 69 amino acid residues of *NEDD4L* and 60 amino acid residues of *MMRN1*. The interaction interface is stabilized by 16 hydrogen bonds and 8 salt Bridges. These structural features suggest that the molecular recognition between *NEDD4L* and *MMRN1* may occur with high affinity and specificity.

**Figure 8:**
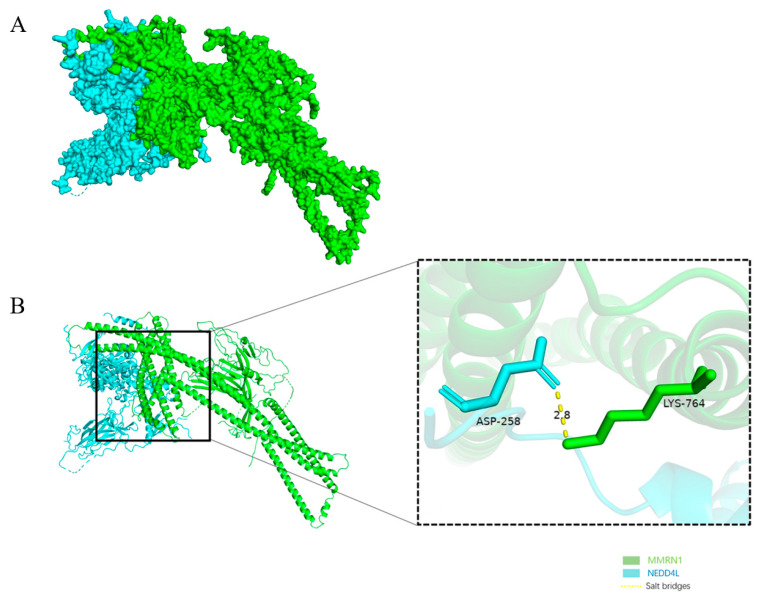
Docking of *MMRN1* with upstream molecules. (**A**) Three-dimensional structure of the target protein–ligand complex; (**B**) Local magnification of the salt bridge interaction structure between *MMRN1* and *NEDD4L*.

Further characterization of the binding interface, Table 3, revealed a complex network of noncovalent interactions. A total of 16 hydrogen bonds were involved in the interface stabilization, among which GLN778, ASN857, and LYS764 residues of *NEDD4L* played a key role. In addition, eight salt Bridges were identified at the interface, such as the ion pair formed between ARG861 of *NEDD4L* and ASP258 of *MMRN1*. These short-range and directed interactions provide the structural basis for the high affinity and specificity of the complex and further provide molecular evidence for the functional hypothesis that *NEDD4L* may regulate *MMRN1* ubiquitination. Its 3D structure is shown in Figure 8.

To verify the specificity of the interaction, a negative control experiment was set up to dock *NEDD4L* with an unrelated protein TRIM47. The control results were significantly different from the main experiment: *MMRN1*-*NEDD4L* complex was significantly better than TRIM47-*NEDD4L* complex in docking score (−351.90), confidence (0.9827) and predicted binding free energy (−10.4 kcal/mol) (score −312.92, confidence 0.9630, *p* < 0.05). Binding free energy −3.8 kcal/mol). Meanwhile, the interaction strength predicted at the TRIM47-*NEDD4L* interface was much weaker than that of the *MMRN1*-*NEDD4L* complex. Detailed results are provided in Supplementary Tables S2–S4 and Supplementary Figure S2. This control result further supports the specificity of the interaction between *MMRN1* and *NEDD4L*.

## 4. Discussion

We developed a prognostic model of E3 ubiquitination in gastric cancer that integrates multiple E3 ligase-related genes and identifies two molecular subtypes (E3GC1/E3GC2), as well as provides a unique approach to risk stratification. While other studies have explored ubiquitination-related models in gastric cancer [28], our work differs from others in that we focus on a broader set of E3 ligases, combined with molecular typing, to provide complementary insights into gastric cancer prognosis. Patients with the E3GC1 subtype exhibited significantly shorter OS (*p* < 0.001). The 10-gene risk signature (represented by *MMRN1*) developed via LASSO-Cox regression demonstrated robust predictive performance across training (TCGA-STAD), internal validation (TCGA-STAD), and external independent (GSE84433) cohorts, markedly outperforming conventional TNM staging. Although the 10-gene prognostic signature we developed showed some predictive power in the training set, it did have more limited performance in the external validation set. This phenomenon indicates that there may be overfitting of the model, and its clinical translation is indeed limited at the current stage. Nevertheless, this model has important value: first, it provides proof of concept for E3 ubiquitin ligase-related genes in gastric cancer prognosis; second, the model successfully classified patients into risk groups with significant survival differences (*p* < 0.001). Finally, it provides an extensible basic framework for subsequent research.

Mechanistic studies indicate that E3 ligases drive the progression of GC. Functional enrichment analysis unveiled that the E3GC1 subtype specifically activates the cell cycle G2/M checkpoint and epithelial–mesenchymal transition (EMT) pathways, accompanied by the activation of immunosuppression-related pathways. The synergistic effect of these pathways may promote tumor proliferation, metastasis, and mediate immune evasion. This finding provides systematic evidence that E3 ligases mediate the malignant progression of GC by regulating cell cycle, EMT, and TIME remodeling.

HR individuals exhibit a unique “immune-exhausted” hot tumor phenotype. Despite widespread activation of 17 immune cell types, encompassing effector memory CD8^+^ T cells and macrophages, regulatory T cells (Tregs) and M2 macrophages are markedly enriched, forming a robust immunosuppressive network. In contrast, the LR group is characterized by retained functional cytotoxic effector cells, such as CD56dim natural killer (NK) cells (*p* < 0.0001), with fewer immunosuppressive components.

Immune infiltration analysis disclosed that HR individuals had a paradoxical TIME feature of high immune cell infiltration but severely suppressed function. This activated but ineffective immune state may drive tumor progression and poor prognosis. Conversely, the LR group, despite lower overall immune infiltration, maintains effective functionality of specific anti-tumor cell types, with relatively scarce or non-dominant immunosuppressive components, contributing to a relatively favorable prognosis.

Drug sensitivity analysis unraveled notable distinctions in anticancer drug responsiveness between risk groups, providing critical evidence for personalized treatment strategies. HR individuals exhibited higher sensitivity to the proteasome inhibitor bortezomib (*p* < 0.001) and the histone deacetylase inhibitor ilisimosib (*p* < 0.01), potentially related to their unique immunosuppressive microenvironment and dysregulated cell cycle control. Conversely, LR individuals were more sensitive to the taxane docetaxel (*p* < 0.001), suggesting that traditional chemotherapy remained effective in this subgroup. Additionally, dynamic monitoring of tumor microenvironment (TME) through ESTIMATE scoring can assist clinicians in adjusting treatment protocols timely, facilitating precision medicine approaches.

As a gene connected with poor prognosis, *MMRN1* is regulated by methylation, protein interactions, and non-coding RNAs (ncRNAs) across various cancers [29]. *MMRN1* facilitates the proliferation and invasion of renal cell carcinoma by activating MMPs via the AMPK pathway [30]. *MMRN1* expression is also tied to the progression of ovarian cancer [31]. In small-cell lung cancer, *MMRN1* interacts with binding immunoglobulins in the endoplasmic reticulum, maintaining ER stress and promoting cisplatin resistance [32]. Our in vitro experiments revealed elevated expression levels of *MMRN1* in GC cell lines (including HGC-27) compared to normal cells. *MMRN1* knockdown markedly suppressed proliferation, inhibited migration, and induced apoptosis in HGC-27 cells.

Molecular docking predicted that *MMRN1*, with high affinity, bound to the E3 ligase *NEDD4L* (ΔG = −10.4 kcal/mol) through a salt bridge (ASP258-LYS764), suggesting that it may be regulated by ubiquitination degradation, thereby affecting the development of GC. This computational prediction aligns with emerging experimental evidence on the role of E3 ligases in GC. For instance, a recent study demonstrated that *NEDD4L* physically interacts with BICC1 via co-immunoprecipitation (Co-IP), promoting its ubiquitination and degradation, which suppresses GC cell proliferation, migration, and EMT processes [33]. Additionally, Wang et al. [34] showed that NEDD4 (a homolog of *NEDD4L*) drives tumor growth in IGF1 signal pathway-dependent GC by modulating the PTEN-IRS1 axis, with clinical data indicating that high NEDD4 and IGF1 co-expression predicts poor prognosis. Although these studies do not directly address *MMRN1*, they underscore the broader significance of E3 ligases in GC progression and provide indirect support for the potential regulation of *MMRN1* by *NEDD4L*. Future work should prioritize experimental validation, such as Co-IP or ubiquitination assays, to confirm the *NEDD4L*-*MMRN1* interaction and its functional impact.

This study established a 10-gene prognostic model. Compared with conventional staging systems, it markedly enhances the accuracy for prognosis prediction and offers a new tool for the risk stratification of GC. Regarding therapeutic strategies, HR individuals are sensitive to the proteasome inhibitor bortezomib and the histone deacetylase inhibitor elicisostat, while taxane-based drugs are more appropriate for LR individuals. In translational medicine, a standardized 10-gene PCR detection kit can be developed to standardize risk stratification. Furthermore, targeting the *NEDD4L*-*MMRN1* interaction to design PROTAC degraders presents a potential targeted therapy strategy. Notably, HR groups exhibit an immune-exhausted phenotype rather than a classical ‘cold’ tumor signature characterized by high immune cell infiltration but functionally suppressed. Therefore, exploring combination regimens of immune checkpoint inhibitors (ICIs) with strategies to reverse immunosuppression (e.g., targeting regulatory T cells [Tregs] or reprogramming M2 macrophages) holds promise for achieving breakthroughs in treatment strategies tailored to the unique immune features of HR patients, demonstrating potential clinical translation value.

However, some limitations should be acknowledged in the current research. First, the absence of in vivo validation, particularly using mouse xenograft models, significantly limits the biological relevance of our findings. Future studies must include animal models to confirm the functional significance of *MMRN1* in tumor growth and metastasis. Second, the external validation using the GSE84433 cohort showed reduced predictive performance (AUC: 0.52–0.60) compared to the TCGA training set (AUC: 0.61–0.71), indicating potential overfitting and limited generalizability. This suggests that our prognostic model may be too optimized for the TCGA dataset’s specific characteristics and requires further refinement in larger, multi-center cohorts to improve its clinical applicability. Third, our functional analyses were conducted primarily in the HGC-27 cell line, which restricts the generalizability of our conclusions to other GC subtypes or cell lines. The reliance on a single-cell model may introduce bias and fail to capture the heterogeneity of GC. Future work should validate these findings in additional cell lines (e.g., AGS, MKN-45) and patient-derived organoids to ensure broader relevance.

## Figures and Tables

**Table 1:** Summary of the top 10 molecular docking models ranked by docking score.

Rank	Docking Score	Confidence Score	Ligand Rmsd (Å)	Interface Residues
1	−351.90	0.9827	87.07	Model 1
2	−305.69	0.9575	77.47	Model 2
3	−301.23	0.9537	100.04	Model 3
4	−284.42	0.9363	74.09	Model 4
5	−266.40	0.9112	74.62	Model 5
6	−263.29	0.9060	104.97	Model 6
7	−261.57	0.9030	99.08	Model 7
8	−259.76	0.8998	79.64	Model 8
9	−254.18	0.8893	109.71	Model 9
10	−251.80	0.8845	85.60	Model 10

Note: The docking score represents the predicted binding affinity. The confidence score estimates the reliability of the predicted pose. Ligand RMSD measures the structural deviation (in Ångstroms) of the docked ligand conformation.

**Table 2:** Key Interaction Parameters between NEDDL4 and *MMRN1* in Model 1.

Parameter	Structure A	Structure B	Interface/Value
Atoms (iNat)	234	228	-
Residues (iNres)	69	60	-
Surface (Å^2^)	93,037	52,113	2343.6
ΔG (kcal/mol)	-	-	−10.4
*p*-value	-	-	0.743 (ns)
Hydrogen bonds (N_HB_)	-	-	16
Salt bridges (N_SB_)	-	-	8

Note: The table summarizes key parameters from the protein–protein interaction analysis. ΔG, binding free energy; ns, not significant (*p* > 0.05).

**Table 3:** Intermolecular interactions between *NEDD4L* and *MMRN1* in Model 1.

**A. Hydrogen Bonds**			
**Structure 1**	**Structure 2**	**Distance (Å)**	**No.**
A:GLN 778[NE2]	B:ARG 250[O]	2.89	1
A:ASN 857[ND2]	B:SER 256[O]	2.42	2
A:LYS 764[NS]	B:GLU 257[O]	3.60	3
A:LYS 764[NS]	B:ASP 258[OD1]	2.85	4
A:ARG 861[NH1]	B:ASP 258[OD2]	3.25	5
A:GLN 817[NE2]	B:ARG 314[O]	3.25	6
A:TYR 545[OH]	B:ASN 510[O]	2.89	7
A:LYS 853[NS]	B:ASP 546[O]	2.49	8
A:ARG 835[NE2]	B:TYR 593[O]	3.29	9
A:ASN 942[ND2]	B:GLU 596[OE1]	2.26	10
A:GLU 515[OE1]	B:ARG 512[NH1]	2.65	11
A:HIS 517[O]	B:HIS 559[NE2]	3.53	12
A:LYS 764[O]	B:ARG 217[NH1]	3.71	13
A:LEU 773[O]	B:ARG 250[NE]	3.66	14
A:LEU 773[O]	B:ARG 250[NE]	3.88	15
A:GLN 778[OE1]	B:ARG 253[NE]	2.22	16
**B. Salt Bridges**			
**Structure 1**	**Structure 2**	**Distance (Å)**	**No.**
A:ARG 861[NE]	B:ASP 258[OD1]	3.90	1
A:LYS 764[NE]	B:GLU 257[OE1]	2.85	2
A:ARG 835[NE]	B:GLU 596[OE2]	3.66	3
A:ARG 861[NE]	B:GLU 515[OE2]	3.25	4
A:GLU 515[OE1]	B:ARG 512[NH2]	2.65	5
A:GLU 515[OE1]	B:LYS 510[NZ]	2.77	6
A:GLU 515[OE2]	B:ARG 512[NH1]	3.62	7
A:GLU 770[OE1]	B:ARG 250[NH1]	2.97	8

Note: Molecular interactions stabilizing the *NEDD4L*–*MMRN1* complex in Model 1. Hydrogen bonds are defined by a donor–acceptor distance < 3.5 Å. Salt bridges are defined by a distance < 4.0 Å between oppositely charged atoms. A total of 16 hydrogen bonds and 8 salt bridges were identified at the protein–protein interface. No disulfide bonds or covalent linkages were detected.

## Data Availability

The original contributions presented in this study are included in the article/supplementary material. Further inquiries can be directed to the corresponding authors.

## Supplementary Materials

The following supporting information can be downloaded at: https://www.mdpi.com/article/10.3390/cimb47110925/s1.

## Abbreviations

The following abbreviations are used in this manuscript:

GC	Gastric Cancer
OS	Overall Survival
UPS	Ubiquitin–Proteasome System
EMT	Epithelial–Mesenchymal Transition
TCGA	The Cancer Genome Atlas
STAD	Stomach Adenocarcinoma
GEO	Gene Expression Omnibus
DEGs	Differentially Expressed Genes
GO	Gene Ontology
KEGG	Kyoto Encyclopedia of Genes and Genomes
GSEA	Gene Set Enrichment Analysis
LASSO	Least Absolute Shrinkage and Selection Operator
KM	Kaplan–Meier
ROC	Receiver Operating Characteristic
AUC	Area Under the Curve
TIME	Tumor Immune Microenvironment
CIBERSORT	Cell-type Identification By Estimating Relative Subsets Of RNA Transcripts
ESTIMATE	Estimation of STromal and Immune cells in MAlignant Tumors using Expression data
ssGSEA	Single-Sample Gene Set Enrichment Analysis
E3GC1/2	E3 Ubiquitin Ligase-associated Gastric Cancer Subtype 1/2
IC50	Half-Maximal Inhibitory Concentration
DMEM	Dulbecco’s Modified Eagle Medium
FBS	Fetal Bovine Serum
siRNA	Small Interfering RNA
RT-qPCR	Quantitative Real-Time Reverse Transcription Polymerase Chain Reaction
SDS-PAGE	Sodium Dodecyl Sulfate–Polyacrylamide Gel Electrophoresis
PVDF	Polyvinylidene Fluoride
HRP	Horseradish Peroxidase
ECL	Enhanced Chemiluminescence
CCK-8	Cell Counting Kit-8
OD	Optical Density
PBS	Phosphate-Buffered Saline
RIPA	Radioimmunoprecipitation Assay Buffer
PMSF	Phenylmethylsulfonyl Fluoride
BCA	Bicinchoninic Acid Assay
ANOVA	Analysis of Variance
FDR	False Discovery Rate
*MMRN1*	Multimerin 1
*NEDD4L*	Neural Precursor Cell Expressed Developmentally Down-regulated 4-Like
PROTAC	Proteolysis-Targeting Chimera
SPR	Surface Plasmon Resonance
Co-IP	Co-Immunoprecipitation
LogFC	Log Fold Change
IQR	Interquartile Range
DCA	Decision Curve Analysis
TME	Tumor Microenvironment
ns	Not Significant
NHB	Number of Hydrogen Bonds
NSB	Number of Salt Bridges
GLOBOCAN	Global Cancer Observatory
HR	Hazard Ratio
E1	Ubiquitin-activating enzyme
E2	Ubiquitin-conjugating enzyme
E3	Ubiquitin ligase
E3s	E3 ubiquitin ligase-associated genes
E3-DEGs	E3-related differentially expressed genes
K-E3DEGs	key prognosis-related E3-related differentially expressed genes
HECW1	HECT, C2 and WW Domain Containing E3 Ubiquitin Protein Ligase 1
FBXW2	F-Box and WD Repeat Domain Containing 2
TRIM25	Tripartite Motif Containing 25
GSVA	Gene Set Variation Analysis
GES-1	Gastric Epithelial cell line-1
AGS	Adenocarcinoma Gastric cell line
MKN-45	Mukai-Noseki-45
MKN-74	Mukai-Noseki-74
HGC-27	Human Gastric Cancer-27
qPCR	Quantitative real-time Polymerase Chain Reaction
GAPDH	Glyceraldehyde-3-Phosphate Dehydrogenase
SYBR	Synthetic Yellow Binding Reagent
TBST	Tris-Buffered Saline with Tween-20
V-FITC	Viability Fluorescein Isothiocyanate
HDOCK	High Ambiguity Driven protein–protein DOCKing
