# Supplementary material for: MMRN1 as a Potential Oncogene in Gastric Cancer: Functional Evidence from In Vitro Studies and Computational Prediction of NEDD4L-Mediated Ubiquitination
Source: Curr Issues Mol Biol. 2025 Nov 6;47(11):925. doi: 10.3390/cimb47110925 (PMC12651045; doi:10.3390/cimb47110925)
Supplement: Supplementary file 1 [file cimb-47-00925-s001.zip › cimb-3905620-supplementary.pdf]

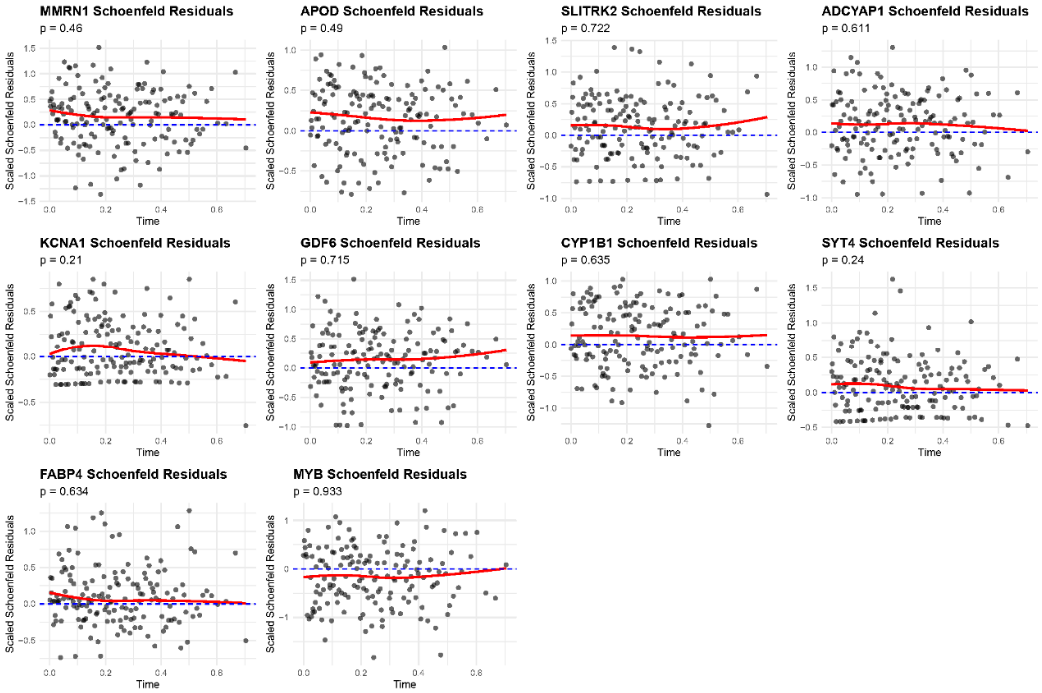

Figure S1. Schoenfeld residual plots for the 10-gene signature in the prognostic model

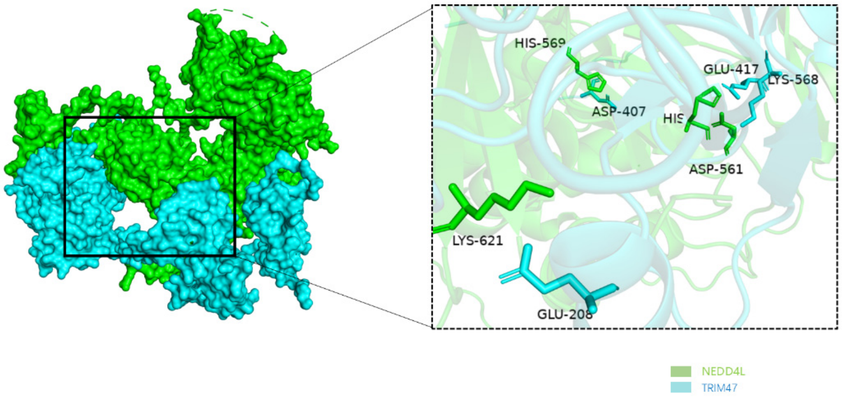

Figure S2. 3D structure and local magnification of NEDD4L docking with TRIM47

Table S1. List of reagents used in this study

Reagent Name	Manufacturer(Catalog Number, dilution)
TRIzol reagent	Invitrogen(99089501)
chloroform	Sinopharm
cDNA synthesis kit	Genebetter Biotechnology(P118-100)
SYBR Green method	Genebetter Biotechnology(P611-50)
MMRN1-specific primers	Tsingke Biotechnology
RIPA lysis buffer	Beyotime(P0013B)
bicinchoninic acid assay kit	Beyotime(P0011)
MMRN1	Proteintech(17878-1-AP， 1:1000)
PBS	Dingguochangsheng(BF0011); Zsbio (ZLI-9062)
separating gel	Genview(GN199-25G)
skim milk powder	Beyotime(P2016)
anti-MMRN1 antibody	Proteintech(17878-1-AP)
antibody dilution buffer	Dingguo Changsheng(AR-0411)
GAPDH anti-body	Proteintech(60004-1)
absolute ethyl alcohol	HuiHong
Protein Marker	Epizyme(WJ102)
acrylamide	Genview(GN199-25G)
Tris-HCl buffer solution at pH 6.8	Beyotime(ST768, ST788)
SDS	Dingguo Changsheng (GS286)
Tris-HCL	BioFroxx(1328GR500)
methyl alcohol	HuiHong
Tween-20	Beyotime(ST825)
TEMED	Beyotime (ST728)
APS	HuiHong(CN.51504)
ECL luminescent liquid	Beyotime(p0018S)
β-actin	Proteintech(20536-1-AP)
Secondary antibody (anti-rabbit)	Proteintech(RGAR001)
Enhanced Cell Viability Assay Kit	Elabscience (E-CK-A362)
Transwell Chamber	Corning(3422)
crystal violet	Shkxbio(C8470)
4% Paraformaldehyde	Biosharp(BL539A)
Annexin V-FITC/PI Apoptosis Detection Kit	Beyotime(C1062L)
0.25% Trypsin	Gibco(C25200056)
PBS	Gibco(C14190500BT)
DMEM Culture Medium	Bio-Channel(BC-M-005)
FBS	Bio-Channel(BC-SE-FBS07)
Lipofectamine 3000	Invitrogen(L3000008)
plasmid	Quanyang

Table S2. HDOCK docking scores and model evaluation for the NEDD4L-TRIM47 complex

Rank	Docking Score	Confidence Score	Ligand rmsd(Å)	Interface residues
1	-312.92	0.9630	108.53	Model 1
2	-304.52	0.9565	73.87	Model 2
3	-300.99	0.9535	83.53	Model 3
4	-299.79	0.9524	108.65	Model 4
5	-295.16	0.9480	130.44	Model 5
6	-276.26	0.9259	91.64	Model 6
7	-267.33	0.9127	97.32	Model 7
8	-263.07	0.9056	102.71	Model 8
9	-262.61	0.9048	106.07	Model 9
10	-259.69	0.8997	78.67	Model 10

Note: The docking score represents the predicted binding affinity. The confidence score estimates the reliability of the predicted pose. Ligand RMSD measures the structural deviation (in Ång-stroms) of the docked ligand conformation.

Table S3. Key Interaction Parameters between NEDDL4 and TRIM47 in Model 1

Parameter	Structure A	Structure B	Interface / Value
Atoms (iNat)	258	245	-
Residues (iNres)	78	69	-
Surface (Å²)	52115	43090	2321.2
ΔG (kcal/mol)	-	-	-3.8
P-value	-	-	0.786 (ns)
Hydrogen bonds (N_{HB})	-	-	15
Salt bridges (N_{SB})	-	-	8

Note: The table summarizes key parameters from the protein-protein interaction analysis. ΔG, binding free energy; ns, not significant ($p > 0.05$).

Table S4. Intermolecular interactions between NEDD4L and TRIM47 in Model 1

A. Hydrogen Bonds

Structure 1	Structure 2	Distance (Å)	No.
A: ARG 101 [NH1]	B: PRO 250 [O]	3.72	1
A: LYS 621 [NZ]	B: GLU 208 [OE2]	3.32	2
A: GLN 865 [NE2]	B: ASP 392 [OD2]	3.70	3
A: HIS 569 [ND1]	B: LEU 408 [O]	3.35	4
A: LYS 572[NZ]	B: LEU 408 [O]	3.26	5
A: ARG 512 [NH1]	B: LEU 414 [O]	2.47	6
A: SER 300 [OG]	B: ARG 250 [NH1]	2.53	7
A: LYS 493 [O]	B: ARG 638 [NE]	2.89	8
A: SER 497 [OG]	B: LYS 425 [NZ]	3.61	9
A: PRO 509 [O]	B: LYS 576 [NZ]	3.48	10
A: ASN 510 [OD1]	B: ARG 574 [NH2]	2.28	11
A: ASP 561 [OD2]	B: LYS 568 [NZ]	3.16	12
A: TYR 660 [OH]	B: ARG 197 [NH1]	2.75	13
A: ASP 705 [OD1]	B: GLN 58 [NE2]	3.36	14
A: TYR 709 [OH]	B: ARG 53 [NH1]	2.15	15

B. Salt Bridges·

Structure 1	Structure 2	Distance (Å)	No.
A: LYS 621 [NZ]	B: GLU 208 [OE2]	3.32	1
A: HIS 569 [ND1]	B: ASP 407 [OD1]	3.95	2
A: HIS 569 [NE2]	B: ASP 407 [OD1]	3.57	3
A: HIS 559 [ND1]	B: GLU 417 [OE1]	2.63	4
A: HIS 559 [NE2]	B: GLU 417 [OE1]	2.08	5
A: HIS 559 [ND1]	B: GLU 417 [OE2]	2.96	6
A: HIS 559 [NE2]	B: GLU 417 [OE2]	3.34	7
A: ASP 561 [OD2]	B: LYS 568 [NZ]	3.16	8

Note: Molecular interactions stabilizing the NEDD4L–MMRN1 complex in Model 1. Hydrogen bonds are defined by a donor–acceptor distance < 3.5 Å. Salt bridges are defined by a distance < 4.0 Å between oppositely charged atoms. A total of 16 hydrogen bonds and 8 salt bridges were identified at the protein–protein interface. No disulfide bonds or covalent linkages were detected.
